# Supplementary material for: The Sequencing-Based Mapping Method for Effectively Cloning Plant Mutated Genes
Source: Int J Mol Sci. 2021 Jun 9;22(12):6224. doi: 10.3390/ijms22126224 (PMC8226582; doi:10.3390/ijms22126224)
Supplement: Supplementary file 1 [file ijms-22-06224-s001.zip › Supplementary Figures -20210603.pdf]

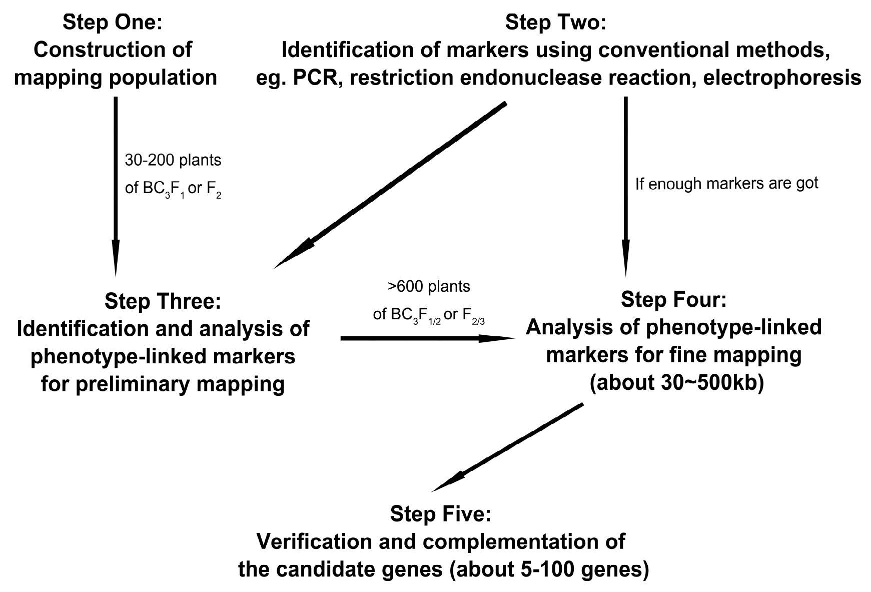

Figure S1. The simplified cloning scheme of the conventional map-based cloning method. Step One: the mutant was crossed with wild type of the other variety to generate mapping population. Step Two: the possible markers were screened out by using the conserved methods like PCR, restriction endonuclease reaction and electrophoresis to get the markers that could be used in the following steps. Step Three: 30~200 heterozygous plants of mapping population were chosen to extract DNA. These DNA samples were used to analyze the marker-indexes for identifying the phenotype-linked markers and the candidate regions. Step Four: if enough markers in the candidate regions that identified in Step Three were screened out, more plants (>600) would be used for the analysis of the recombinants to narrow the candidate region. If the markers were not enough and the candidate regions were too large, another mapping population would be needed. Step Five: to identify and confirm the mutated sites, the sequences of the candidate regions were amplified using PCR to be sequenced and the candidate genes were transformed into the mutants to complement their phenotypes.

Figure S2. The simplified cloning scheme for the homologous mutant. Step One: the homologous mutant was crossed with wild type of the other variety to generate the F_2 population. Step Two: 30~200 heterozygous plants of F_2 population were chosen to extract DNA, the DNA samples were mixed with equimolar of each, re-sequenced, and analyzed. The markers (mainly SNP and In-Del) were identified and analyzed. Their marker-indexes were analyzed to identify the high-quality markers and the candidate regions using the MutMap method. Step Three: the F_2 population was used for the analysis of the recombinants to identify and narrow the candidate region with the markers which were downloaded from the sharing platform on the internet or identified from the re-sequencing results. Step Four: using the re-sequencing genome data, the mutational site (the △marker-index was about 1) in the target region were identified.

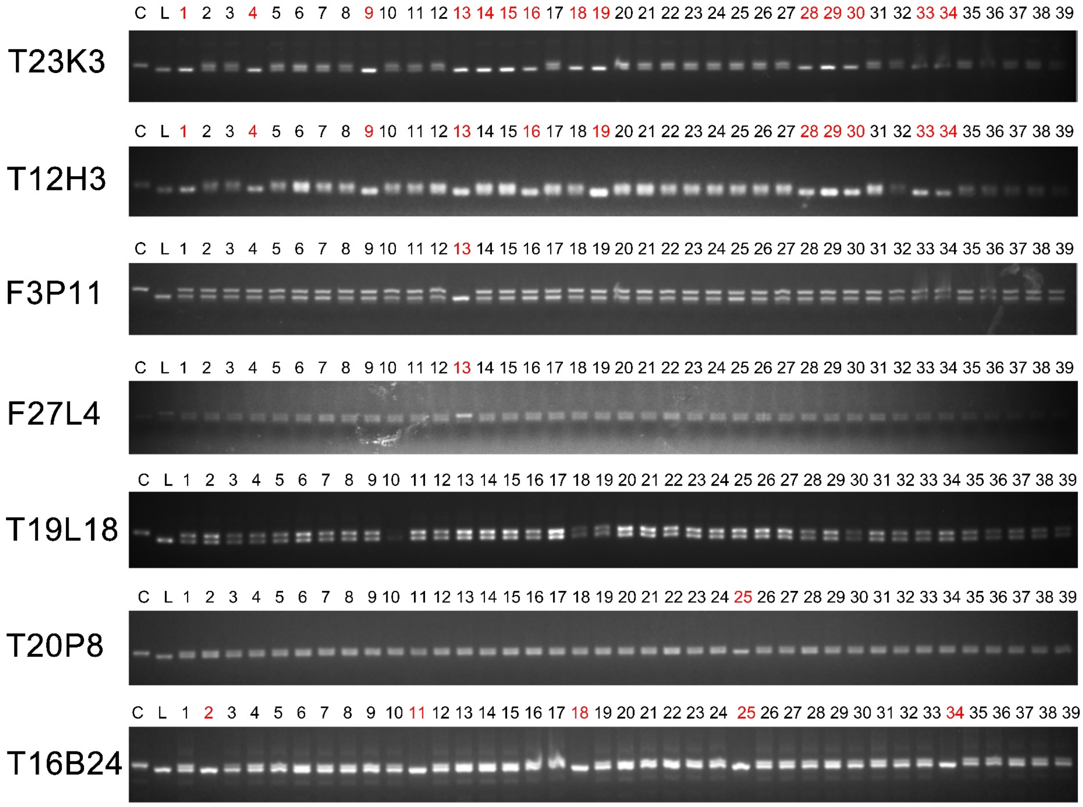

Figure S3. The PCR results of some SSLP markers in the BC_2F_1 population of *275-3*. The markers were showed at the left side, and the red numbers indicated the recombinants. T23K3, T12H3, F3P11, F27L4, T20P8 and T16B24 are located around Chr2: 388,035 bp, Chr2: 2,584,861 bp, Chr2: 8,410,147 bp, Chr2: 10,068,751 bp, Chr2: 11,595,846 bp and Chr2: 16,339,214 bp respectively. C, Col; L, L*er*.

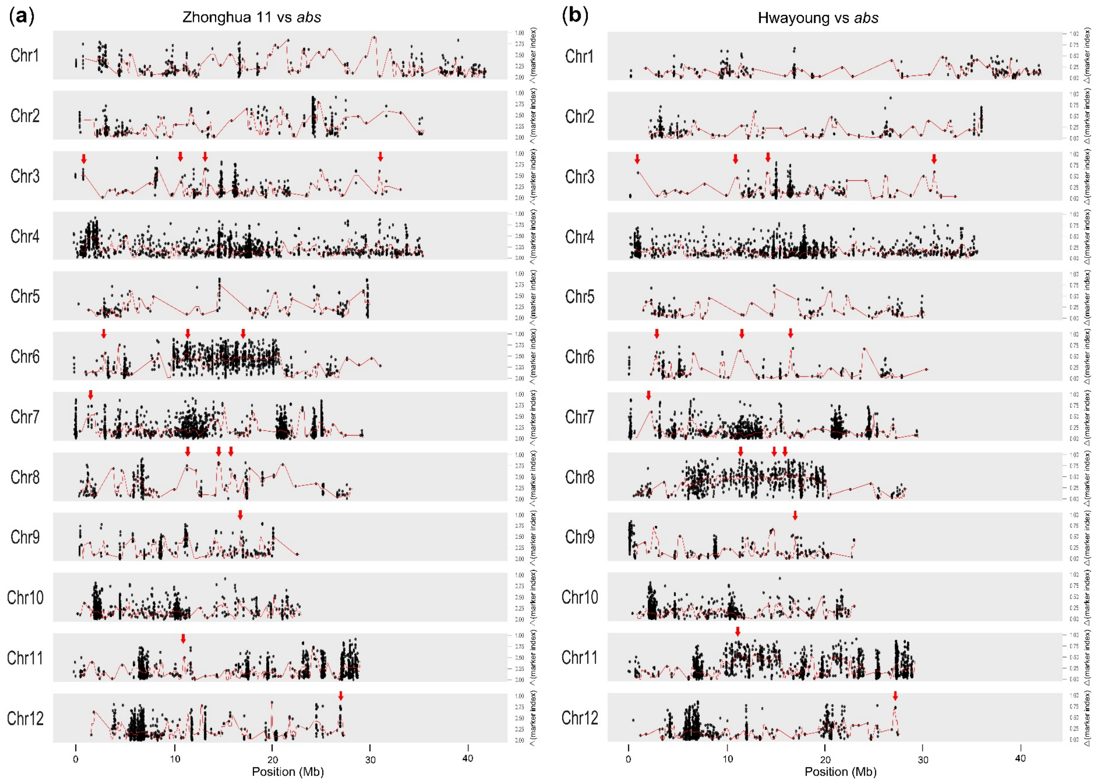

Figure S4. The △marker-index plots of the mutant *abs* generated by the MutMap method. The △marker-index plots were analyzed based on Zhonghua11 (a) and Hwayoung (b) respectively. The red lines represent the sliding window average △ marker-index values of the 500 Kb interval with 100 kb increments. And the combination of them showed that there 14 candidate regions in 7 chromosomes with the threshold 0.5 (the red arrow indicated).

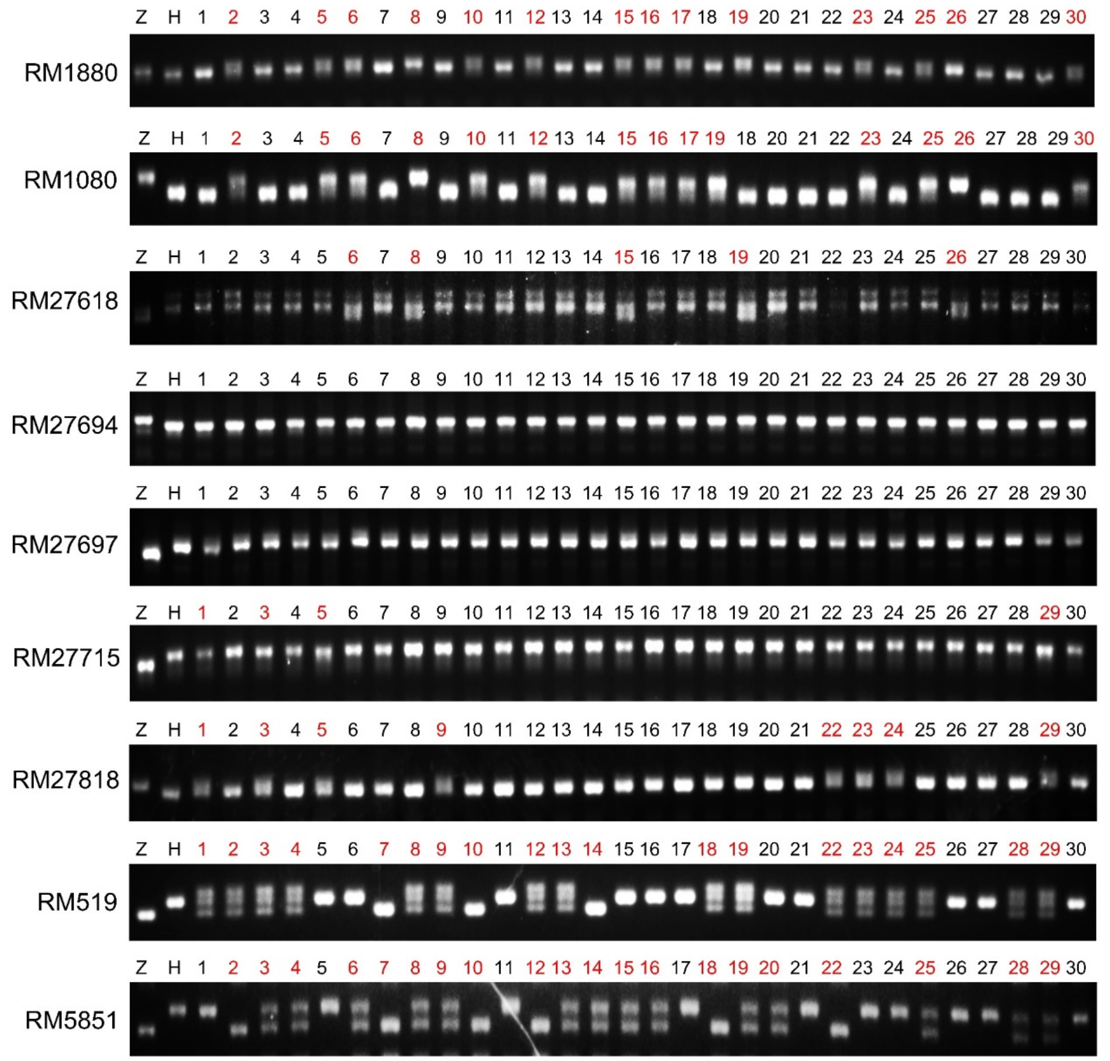

Figure S5. The PCR results of some SSR markers in the F_2 population of rice mutant *abs*. The markers were showed at the left side, and the red numbers indicated the recombinants. RM1880, RM1080, RM27618, RM27694, RM27697 RM27715, RM27818, RM519 and RM5851 are located around Chr12: 747,262 bp, Chr12: 906,291 bp, Chr12: 3,804,685 bp, Chr12: 4,814,750 bp, Chr12: 4,920,770 bp, Chr12: 5,189,063 bp, Chr12: 7,567,862 bp, Chr12: 19,932,442 bp and Chr12: 26,186,010 bp respectively. Z, Zhonghua 11; H, Hwayoung.

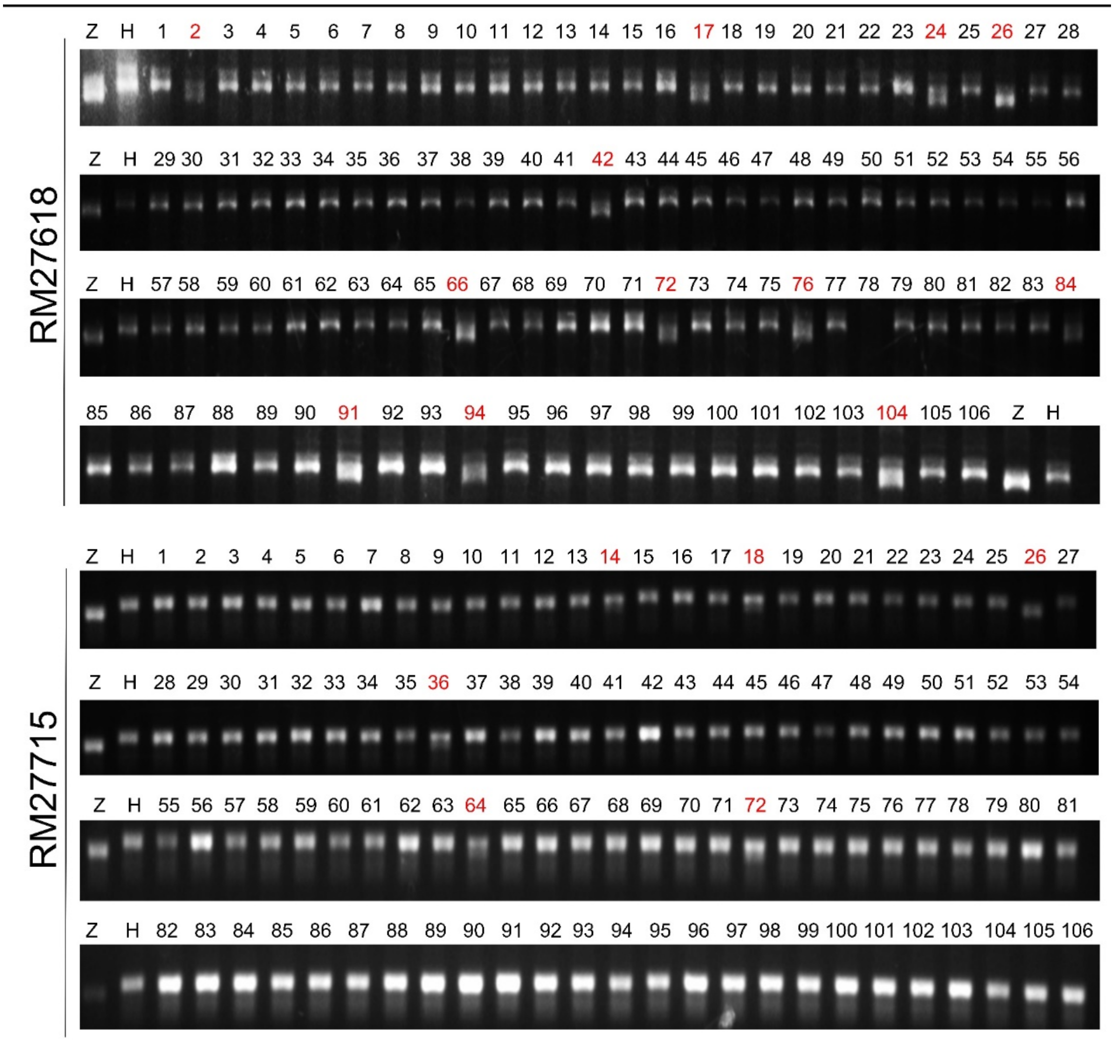

Figure S6. The PCR results of RM27618 and RM27715 in the F_2 population of rice mutant *abs*. The markers, RM27618 (Chr12: 3,804,685 bp) and RM27715 (Chr12: 5,189,063 bp), were showed at the left side, and the red numbers indicated the recombinants. Z, Zhonghua 11; H, Hwayoung.
